# Supplementary material for: GANterfactual—Counterfactual Explanations for Medical Non-experts Using Generative Adversarial Learning
Source: Front Artif Intell. 2022 Apr 8;5:825565. doi: 10.3389/frai.2022.825565 (PMC9024220; doi:10.3389/frai.2022.825565)
Supplement: Supplementary file 1 [file Data_Sheet_1.PDF]

Supplementary Material

1 CLASSIFIER ARCHITECTURE

Layer	Description	Number of Filters	Size	Stride	Dropout Probability
1	Conv2D	96	11 x 11	4	-
2	MaxPooling2D	-	2 x 2	2	-
3	Batch Normalization	-	-	-	-
4	Conv2D	256	11 x 11	1	-
5	MaxPooling2D	-	2 x 2	2	-
6	Batch Normalization	-	-	-	-
7	Conv2D	384	3 x 3	1	-
8	Batch Normalization	-	-	-	-
9	Conv2D	384	3 x 3	1	-
10	Batch Normalization	-	-	-	-
11	Conv2D	256	3 x 3	1	-
12	MaxPooling2D	-	2 x 2	2	-
13	Batch Normalization	-	-	-	-
14	Flatten	-	-	-	-
15	Dense	-	4096	-	-
16	Dropout	-	-	-	0.4
17	Batch Normalization	-	-	-	-
18	Dense	-	4096	-	-
19	Dropout	-	-	-	0.4
20	Batch Normalization	-	-	-	-
21	Dense	-	1000	-	-
22	Dropout	-	-	-	0.4
23	Batch Normalization	-	-	-	-
24	Dense	-	2	-	-

Table S1. L2 bias and kernel regularization with a regularization factor of 0.001 was applied to all convolutional and dense layers except layer 25.

2 STUDY DESIGN

The following figures show the online study that was conducted. The condition of our counterfactual approach is depicted. The other conditions, i.e. LIME and LRP, were designed analogously.

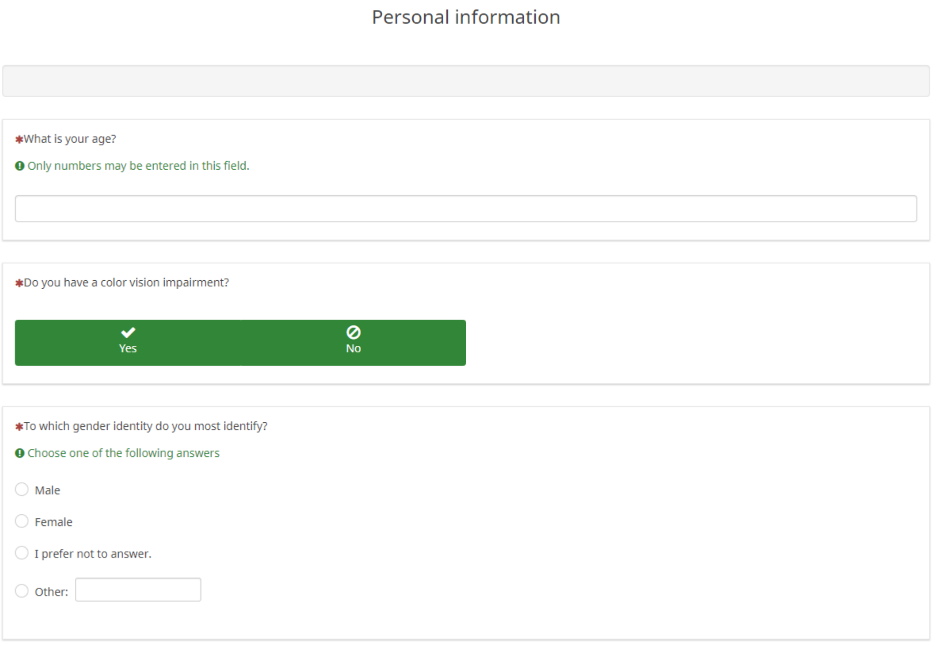

Figure S1. First page of our online study, Part 1

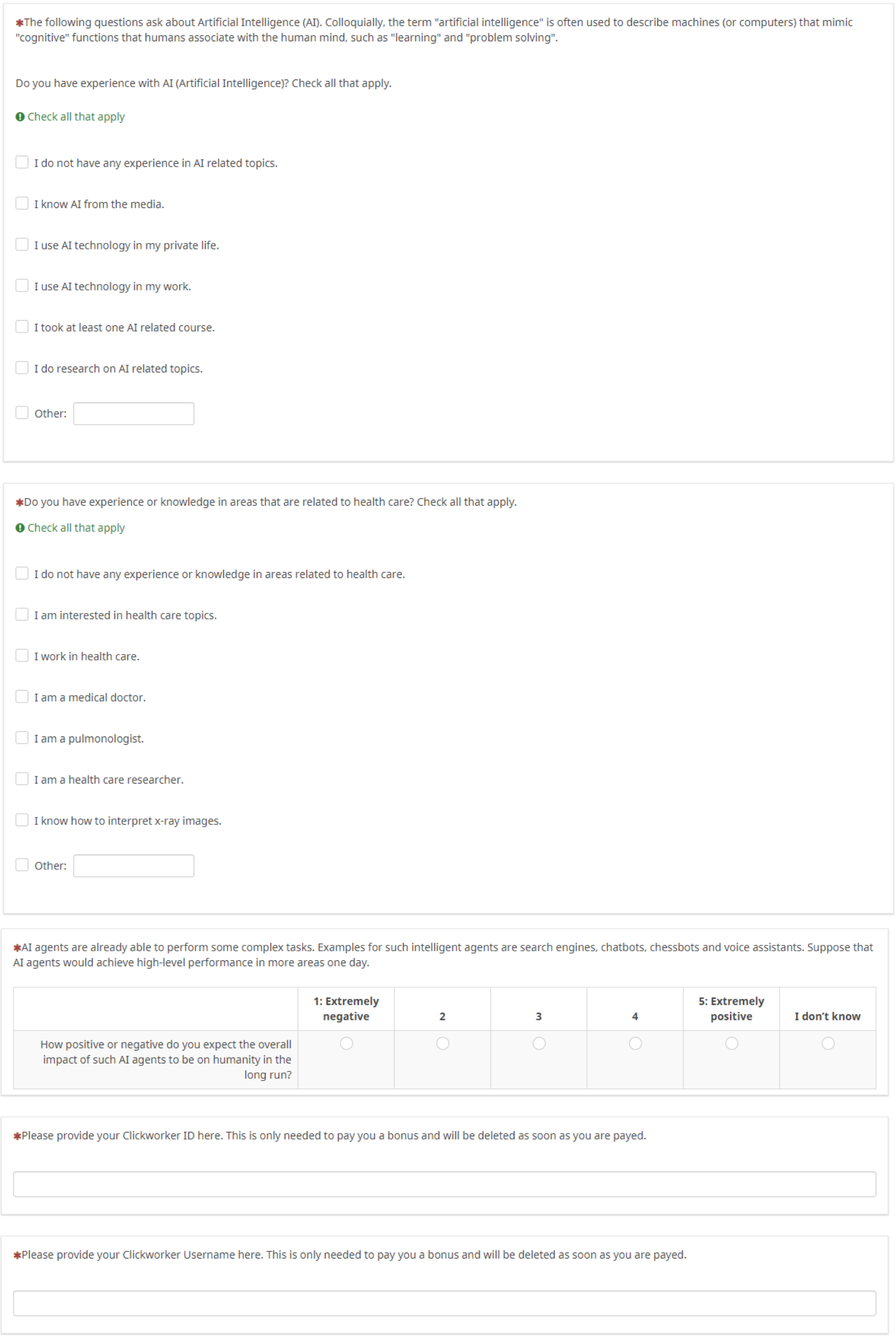

*The following questions ask about Artificial Intelligence (AI). Colloquially, the term "artificial intelligence" is often used to describe machines (or computers) that mimic "cognitive" functions that humans associate with the human mind, such as "learning" and "problem solving".

Do you have experience with AI (Artificial Intelligence)? Check all that apply.

Check all that apply

- I do not have any experience in AI related topics.
- I know AI from the media.
- I use AI technology in my private life.
- I use AI technology in my work.
- I took at least one AI related course.
- I do research on AI related topics.
- Other:

*Do you have experience or knowledge in areas that are related to health care? Check all that apply.

Check all that apply

- I do not have any experience or knowledge in areas related to health care.
- I am interested in health care topics.
- I work in health care.
- I am a medical doctor.
- I am a pulmonologist.
- I am a health care researcher.
- I know how to interpret x-ray images.
- Other:

*AI agents are already able to perform some complex tasks. Examples for such intelligent agents are search engines, chatbots, chessbots and voice assistants. Suppose that AI agents would achieve high-level performance in more areas one day.

	1: Extremely negative	2	3	4	5: Extremely positive	I don't know
How positive or negative do you expect the overall impact of such AI agents to be on humanity in the long run?	○	○	○	○	○	○

*Please provide your Clickworker ID here. This is only needed to pay you a bonus and will be deleted as soon as you are payed.

*Please provide your Clickworker Username here. This is only needed to pay you a bonus and will be deleted as soon as you are payed.

Figure S2. First page of our online study, Part 2.

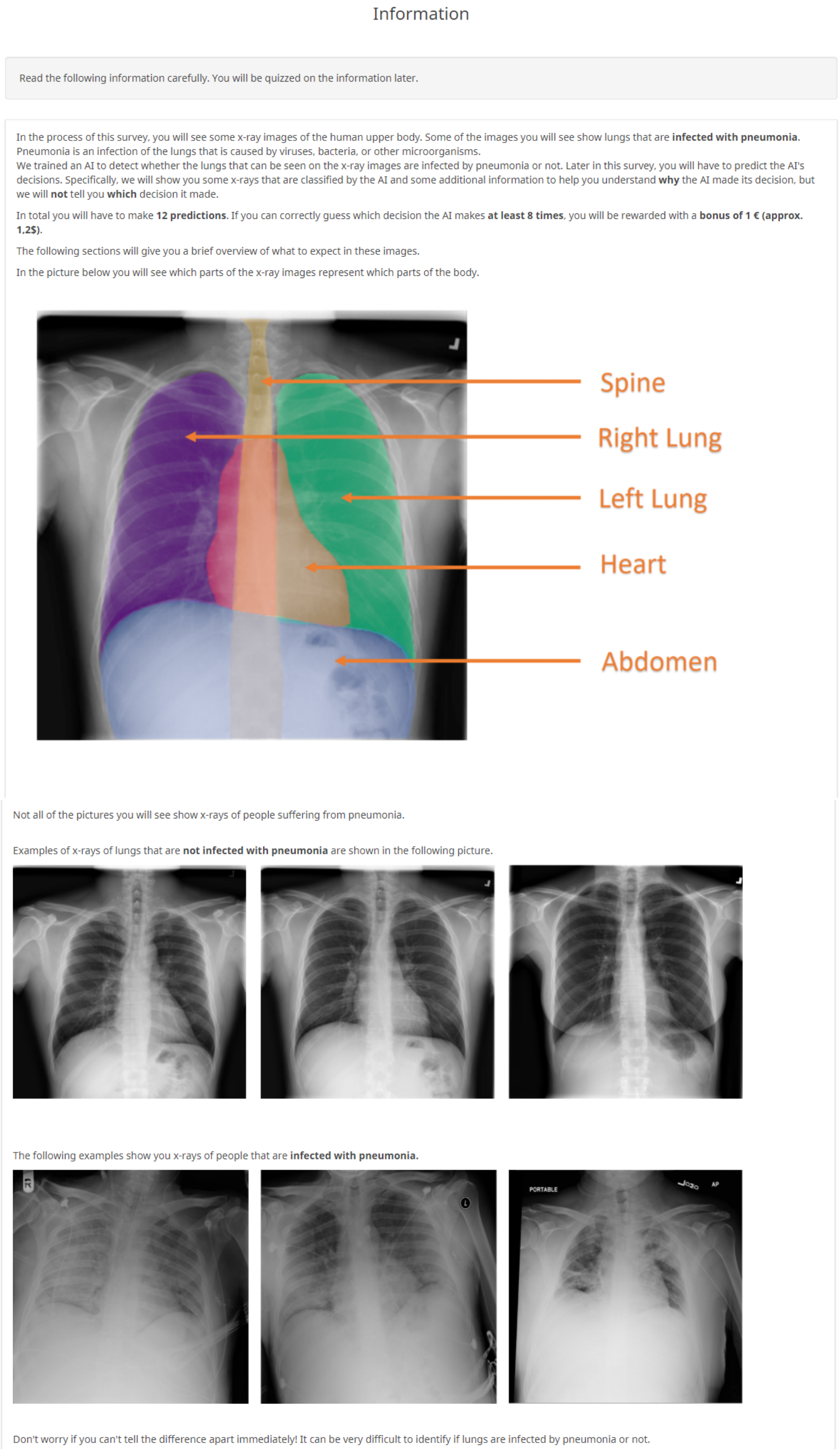

Information

Read the following information carefully. You will be quizzed on the information later.

In the process of this survey, you will see some x-ray images of the human upper body. Some of the images you will see show lungs that are **infected with pneumonia**. Pneumonia is an infection of the lungs that is caused by viruses, bacteria, or other microorganisms.
We trained an AI to detect whether the lungs that can be seen on the x-ray images are infected by pneumonia or not. Later in this survey, you will have to predict the AI's decisions. Specifically, we will show you some x-rays that are classified by the AI and some additional information to help you understand **why** the AI made its decision, but we will **not** tell you **which** decision it made.

In total you will have to make **12 predictions**. If you can correctly guess which decision the AI makes **at least 8 times**, you will be rewarded with a **bonus of 1 € (approx. 1,2$)**.

The following sections will give you a brief overview of what to expect in these images.

In the picture below you will see which parts of the x-ray images represent which parts of the body.

Not all of the pictures you will see show x-rays of people suffering from pneumonia.

Examples of x-rays of lungs that are **not infected with pneumonia** are shown in the following picture.

The following examples show you x-rays of people that are **infected with pneumonia**.

Don't worry if you can't tell the difference apart immediately! It can be very difficult to identify if lungs are infected by pneumonia or not.

Figure S3. Second page of our online study, Part 1.

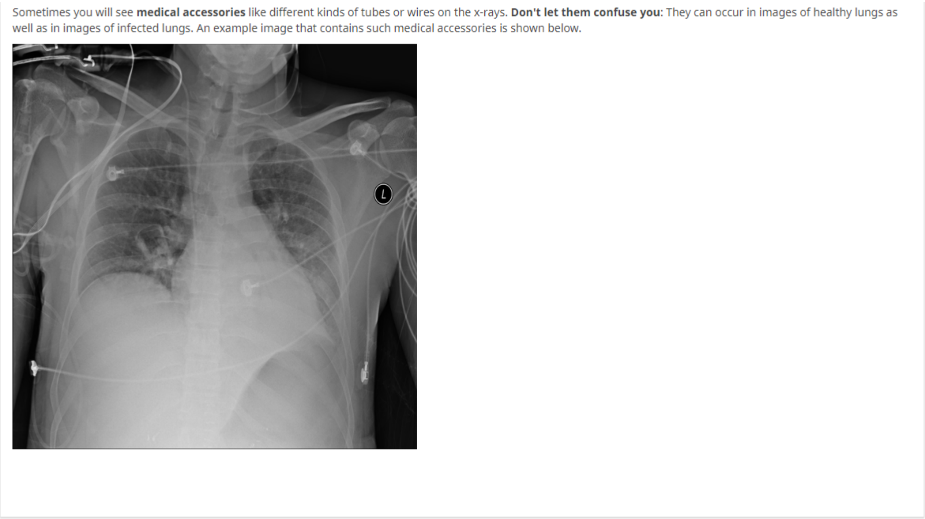

Figure S4. Second page of our online study, Part 2.

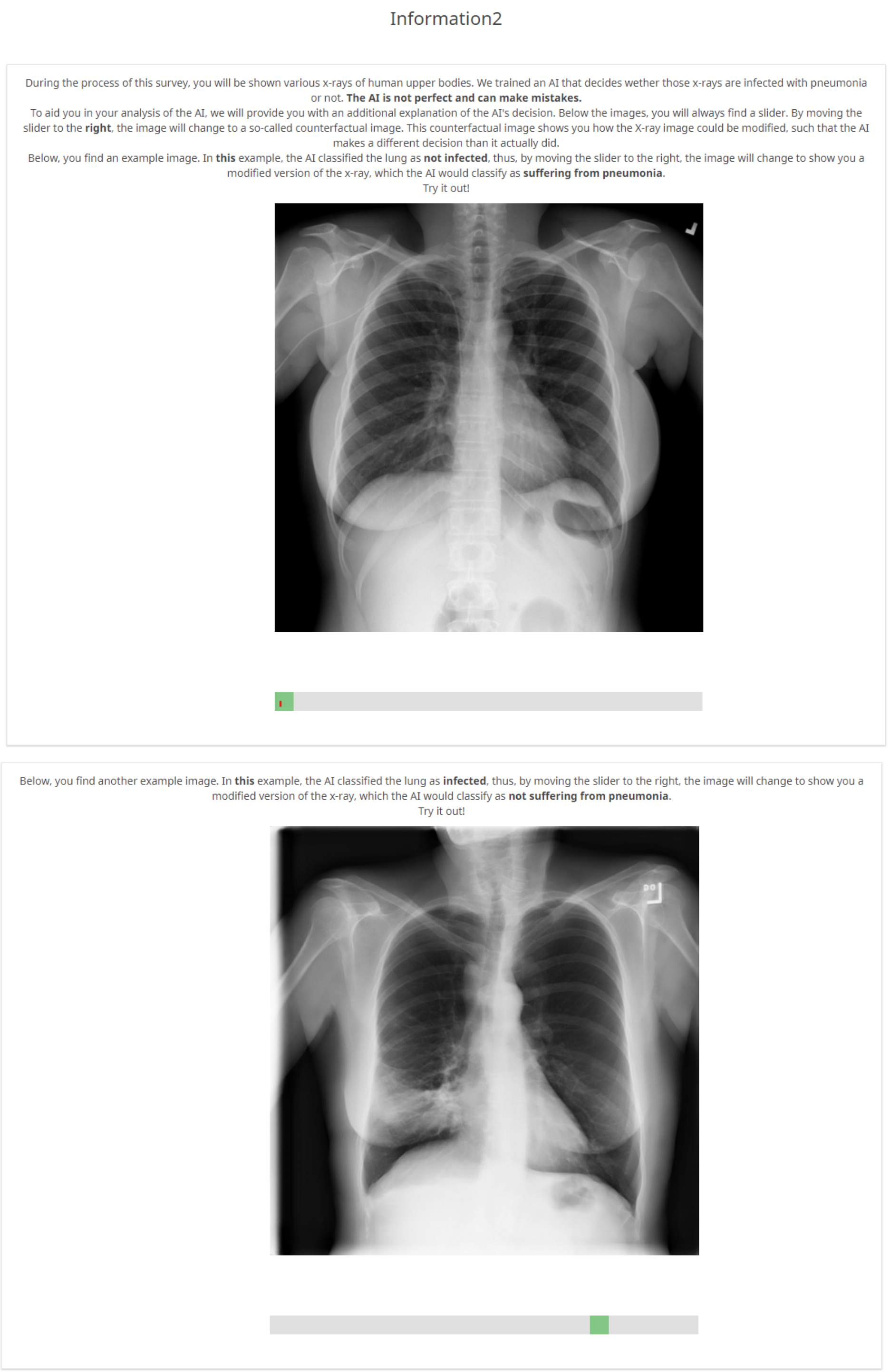

Figure S5. Third page of our online study.

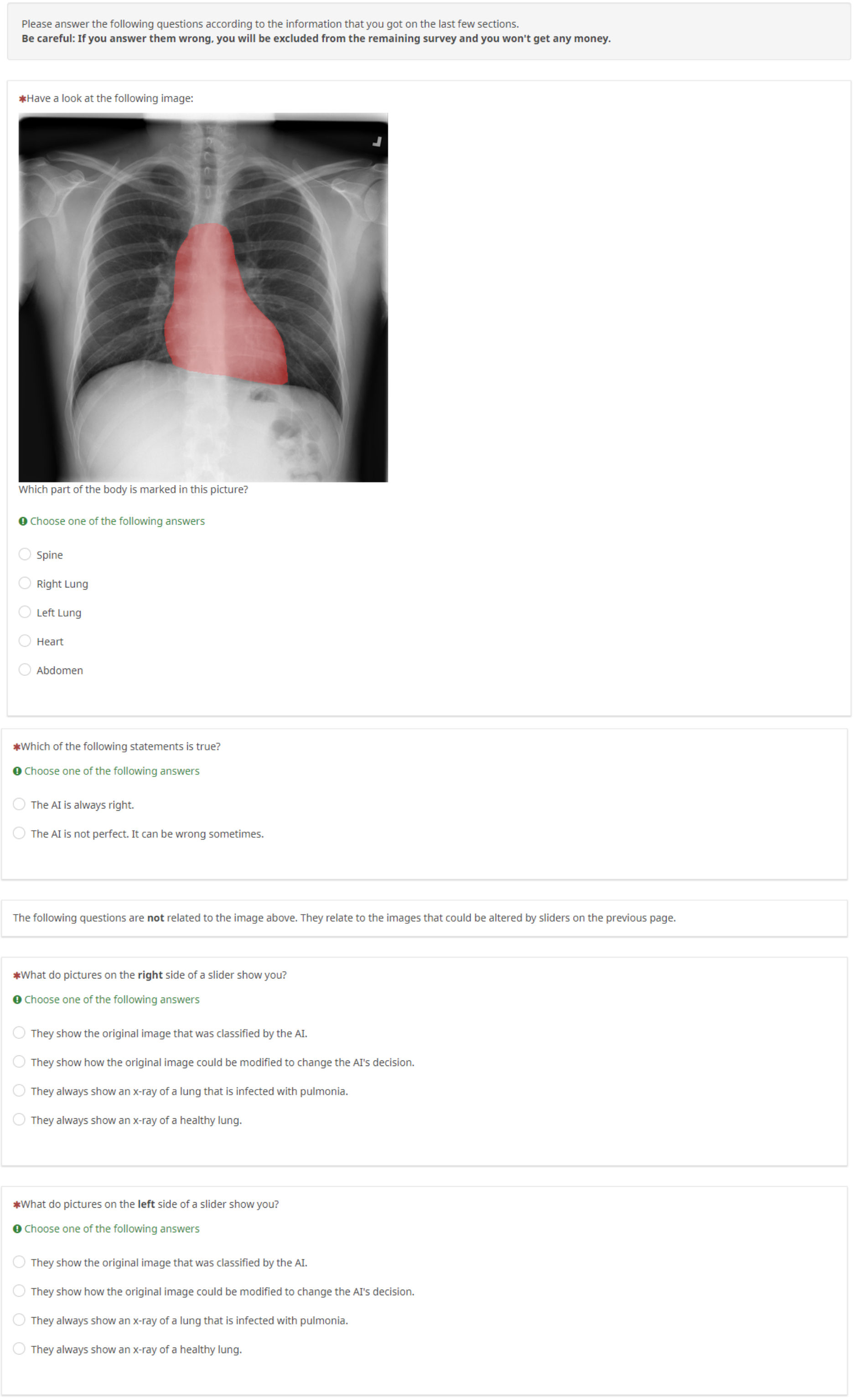

Figure S6. Qualification test (Quiz) of our online study.

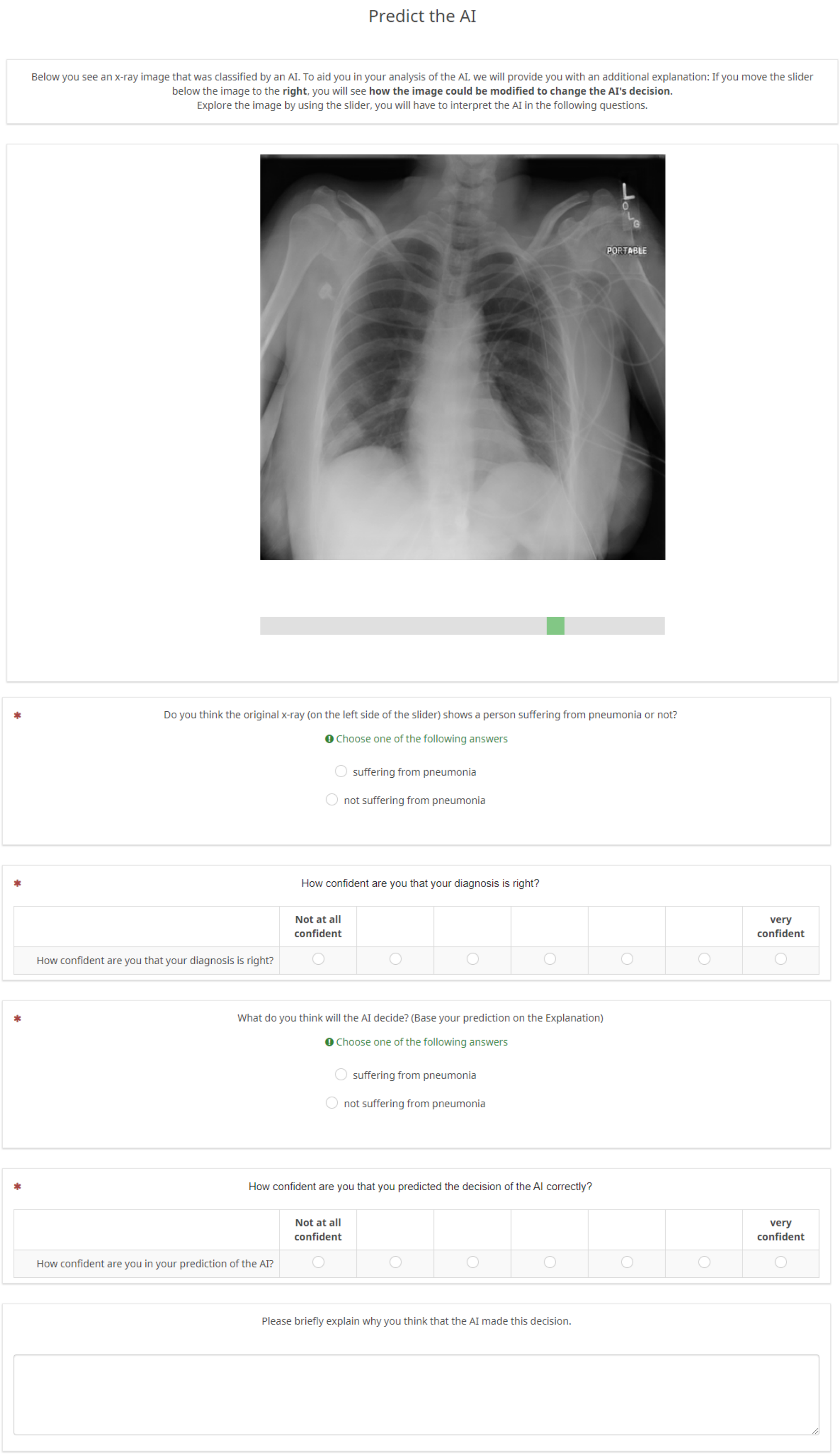

Predict the AI

Below you see an x-ray image that was classified by an AI. To aid you in your analysis of the AI, we will provide you with an additional explanation: If you move the slider below the image to the **right**, you will see **how the image could be modified to change the AI's decision**.
Explore the image by using the slider, you will have to interpret the AI in the following questions.

* Do you think the original x-ray (on the left side of the slider) shows a person suffering from pneumonia or not?

Choose one of the following answers

- suffering from pneumonia
- not suffering from pneumonia

* How confident are you that your diagnosis is right?

	Not at all confident					very confident
How confident are you that your diagnosis is right?	○	○	○	○	○	○	○

* What do you think will the AI decide? (Base your prediction on the Explanation)

Choose one of the following answers

- suffering from pneumonia
- not suffering from pneumonia

* How confident are you that you predicted the decision of the AI correctly?

	Not at all confident					very confident
How confident are you in your prediction of the AI?	○	○	○	○	○	○	○

Please briefly explain why you think that the AI made this decision.

Figure S7. Exemplary prediction task of our online study.

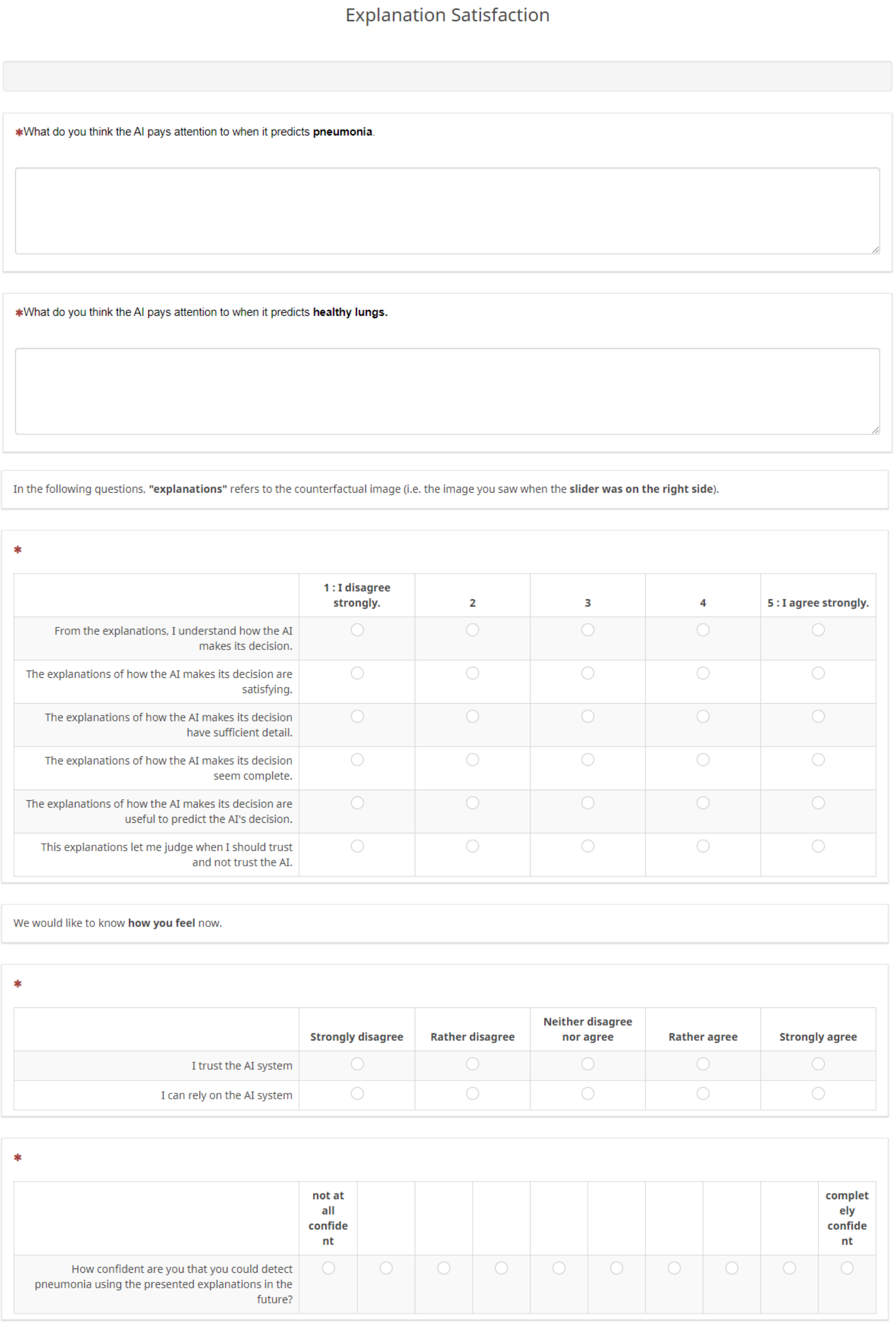

Explanation Satisfaction

*What do you think the AI pays attention to when it predicts **pneumonia**.

*What do you think the AI pays attention to when it predicts **healthy lungs.**

In the following questions, **"explanations"** refers to the counterfactual image (i.e. the image you saw when the **slider was on the right side**).

*

	1 : I disagree strongly.	2	3	4	5 : I agree strongly.
From the explanations, I understand how the AI makes its decision.					
The explanations of how the AI makes its decision are satisfying.					
The explanations of how the AI makes its decision have sufficient detail.					
The explanations of how the AI makes its decision seem complete.					
The explanations of how the AI makes its decision are useful to predict the AI's decision.					
This explanations let me judge when I should trust and not trust the AI.					

We would like to know **how you feel** now.

*

	Strongly disagree	Rather disagree	Neither disagree nor agree	Rather agree	Strongly agree
I trust the AI system					
I can rely on the AI system					

*

	not at all confide nt									complet ely confide nt
How confident are you that you could detect pneumonia using the presented explanations in the future?										

Figure S8. Concluding questions of our online study, Part 1.

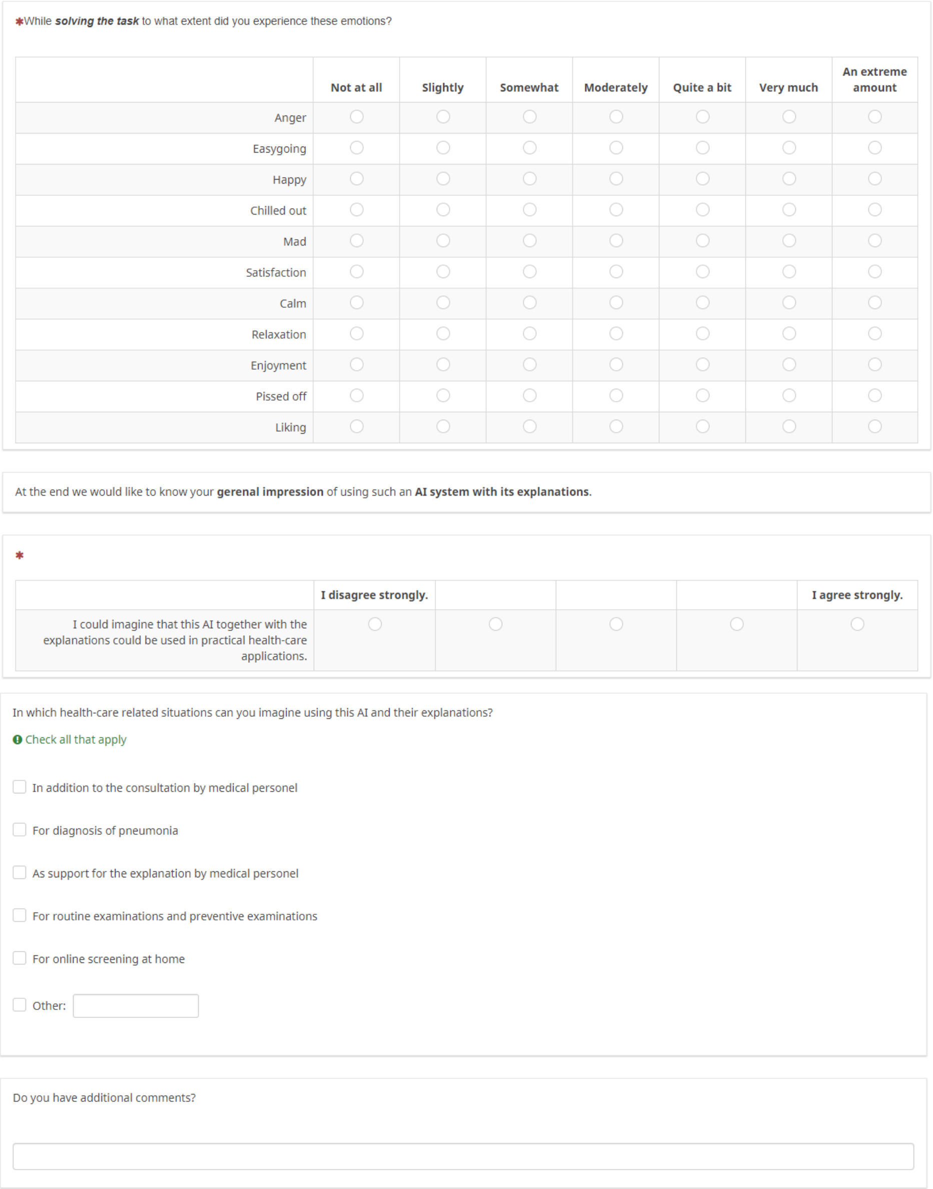

*While ***solving the task*** to what extent did you experience these emotions?

	Not at all	Slightly	Somewhat	Moderately	Quite a bit	Very much	An extreme amount
Anger	○	○	○	○	○	○	○
Easygoing	○	○	○	○	○	○	○
Happy	○	○	○	○	○	○	○
Chilled out	○	○	○	○	○	○	○
Mad	○	○	○	○	○	○	○
Satisfaction	○	○	○	○	○	○	○
Calm	○	○	○	○	○	○	○
Relaxation	○	○	○	○	○	○	○
Enjoyment	○	○	○	○	○	○	○
Pissed off	○	○	○	○	○	○	○
Liking	○	○	○	○	○	○	○

At the end we would like to know your **gerenal impression** of using such an **AI system with its explanations**.

*

	I disagree strongly.				I agree strongly.
I could imagine that this AI together with the explanations could be used in practical health-care applications.	○	○	○	○	○

In which health-care related situations can you imagine using this AI and their explanations?

Check all that apply

- ☐ In addition to the consultation by medical personel
- ☐ For diagnosis of pneumonia
- ☐ As support for the explanation by medical personel
- ☐ For routine examinations and preventive examinations
- ☐ For online screening at home
- ☐ Other:

Do you have additional comments?

Figure S9. Concluding questions of our online study, Part 2.
